# Effect of High-Pressure Processing on Color, Texture and Volatile Profile During Sardine Refrigeration

**DOI:** 10.3390/foods14020329

**Published:** 2025-01-20

**Authors:** Lama Ismaiel, Ancuta Nartea, Benedetta Fanesi, Paolo Lucci, Deborah Pacetti, Henry Jaeger, Felix Schottroff

**Affiliations:** 1 Department of Agricultural, Food and Environmental Sciences, Università Politecnica delle Marche, 60131 Ancona, Italy; l.ismaiel@univpm.it (L.I.); a.nartea@univpm.it (A.N.); b.fanesi@pm.univpm.it (B.F.); d.pacetti@univpm.it (D.P.); 2 Institute of Food Technology, BOKU University, Muthgasse, 18, 1190 Vienna, Austria; henry.jaeger@boku.ac.at (H.J.); felix.schottroff@boku.ac.at (F.S.)

**Keywords:** fresh sardine, nonthermal, emerging technology, cold storage, shelf life, inactivation

## Abstract

Extending sardine shelf life while maintaining their quality is challenging even with non-thermal technologies like high-pressure processing (HPP). This study examines the effects of HPP at 400 and 600 MPa for holding times of 1, 2.5, 5, and 10 min on fresh sardines during 14 days of cold storage. Physicochemical attributes, including texture, color, and volatile organic profiles, were assessed. Increasing both pressure and holding times resulted in increased levels of hardness, chewiness, and L* during storage. HPP-treated samples maintained lower a* values compared to the control ones by the end of the experiment. The volatile profile of HPP samples was significantly affected compared to control samples, which developed exclusively volatile oxidation compounds (hexanal and 2,4-hexadienal) by the end of the storage. Volatile groups such as aldehyde and ketone were slightly impacted by both storage and HPP treatments (i.e., pressure and holding time). Ketone levels were consistently lower in all treated samples, ranging from 25.3% to 33.6% at 400 MPa and 600 MPa, respectively, compared to the control samples, which had a ketone level of 40.5% on day 14. These findings indicate the potential of HPP in prolonging shelf life and preserving quality in the sardine market.

## 1. Introduction

Sardines are recognized as a valuable and economically accessible source of essential nutrients worldwide. In the Mediterranean Sea, *S. pilchardus* is the most relevant pelagic fish [1]. Although comprehensive data on pelagic fish consumption across the entire European region is limited, it is well-documented that, for instance, the average person in Spain consumed 0.41 kg of fresh sardines in 2021 [2]. Sardines’ nutritional value is provided by essential lipids and easily digestible proteins packed with minor nutrients such as vitamins (i.e., vitamin E), and minerals, promoting a balanced human diet. Sardines are an excellent source of heart-healthy omega-3 fatty acids, including eicosapentaenoic acid (EPA) and docosahexaenoic acid (DHA) [3]. Depending on their reproductive stage, sardines captured during the spawning period exhibited considerable levels of the EPA/DHA ratio compared to those in other reproductive stages [4]. A study suggests that consuming 10 g of sardines caught during autumn or 100 g of sardines collected in winter would be sufficient to meet the recommended daily intake of omega-3 fatty acids [5]. However, raw sardines’ flesh, which possesses a high water activity, is a very perishable food product with a short shelf life of around 3–4 days at cold storage (4 °C), which is exposed to the microbial spoilage, protein denaturation, and lipid oxidation of the long-chain polyunsaturated fatty acids (PUFA) occurring sequentially or simultaneously [6]. Some lipid oxidation products (e.g., aldehydes) were linked to the denaturation of metmyoglobin [7,8]. Discoloration of the sardine flesh stored in ice at 4 °C for 15 days was associated with oxidation and the denaturation of myoglobin [9]. The concentration of odor-active compounds associated with oxidized flavors increased over 9 days of storage at 4 °C. This observation was attributed to the increased levels of rancid odor in sardines [10]. Extending the shelf life of fresh sardines while maintaining their nutritional quality (i.e., protein and lipid) and the physicochemical parameters (i.e., color, sensory, and texture traits) is necessary. Conventional methods including chilling, salting, canning, fermenting, and freezing are not optimal solutions, since various limitations have been reported [11]. Frozen fish exhibited undesirable alterations in both color and texture. Canning, for example, does not maintain the freshness of fish. However, this goal might now be feasible thanks to the advancement in emerging technologies such as high-pressure processing (HPP).

HPP is a non-thermal technology that has been successfully employed for various food items, and it is increasingly being recognized as a promising minimal processing technique in the seafood industry [12]. This treatment involves placing packaged products inside a high-pressure chamber, where they are subjected to pressures of up to 600 MPa to effectively eliminate major seafood pathogens [11]. The scientific opinion of the EFSA panel reported that pressures between 400 and 600 MPa applied for a few minutes are typically used to ensure food safety within the industrial setting [13]. For example, the biological hazard of *Staphylococcus aureus* was eliminated using 600 MPa for 8 min, 550 MPa for 10 min, and 500 MPa for 15 min [13]. There is a consensus on using pressure levels up to 600 MPa for <15 min when treating various types of fish, such as shrimp, salmon, cod, herrings, mackerel, and rainbow trout [14,15,16,17].

Common seafood infections like *Vibrio* and *Listeria* spp. are eliminated by applying HPP (400–600 MPa), which also inhibits the growth of spoilage microorganisms [11]. Moreover, HPP at 450 and 600 MPa for 5 min provided superior textural properties of tuna loins (*Katsuwonus pelamis*) in terms of hardness and springiness when compared to other processes such as steam cooking [18]. Furthermore, the water-holding capacity decreased from 57 to 44% when tuna loins were treated at 600 MPa for 5 min, which suggests the potential of HPP to be used as an alternative to the tuna precooking process, as it increased the yield [18].

Pressure values at 450 and 600 MPa were used to process snow crab (*Chionoecetes opilio*) for 2 min to create a chromatic value for melanosis determination, using digital image analysis for a quick assessment of the product appearance [19]. The European hake (*Merluccius merluccius*) after HPP at 150 and 450 MPa for 2 min, became more resilient and springier, and displayed improved sensory qualities when it was turned into fishcakes [20]. The application of high-pressure treatment at 300 MPa for a short time (2 min) extended the shelf life of oysters from 6 to 8 days to 12 days [21]. A 5 min HPP treatment at 600 MPa decreased microbial growth and increased the shelf life of sea bass filets by 60 days during cold storage at 4 °C [22]. Additionally, HPP technology has contributed to improving fish texturization and facilitating 100% meat separation from shellfish items [23,24]. The sensory evaluation of oysters subjected to HPP at 300 MPa for 2 min, including texture, color, and odor, exhibited a significantly slower decline and remained within acceptable limits for up to 12 days [24]. In contrast, the sensory records for untreated pacific oysters deteriorated rapidly and became unacceptable as early as day 4 [21]. No differences in the overall appearance and odor of smoked cod were reported after HPP at 400, 500, and 600 MPa for 5 and 10 min [15]. This suggests that HPP treatment does not cause any observable sensory drawbacks, and further advancements in improving the quality of stored seafood using high pressure were reported. HPP treatment at 200 and 300 MPa for 5 min reduced oxidative processes and lipid oxidation in haddock (*Melanogrammus aeglefinus*) and mackerel (*Scomber scombrus*) fish during frozen storage due to the inactivation of pro-oxidative enzymes such as lipoxygenases and peroxidases [25]. The inhibition of cholesterol and polyunsaturated fatty acid (PUFA) oxidation in herrings and mackerel was achieved after HPP treatment at 600 MPa for 10 min [14]. The application of HPP at 150 and 200 MPa for 3 min to barramundi (*Lates calcarifer*) muscle effectively postponed lipid oxidation throughout frozen storage for more than 4 months [26]. Enhanced quality attributes of hilsa (*Tenualosa ilisha*) filets, including texture, reduced levels of free fatty acids (FFAs), and minimized lipid oxidation were reported after HPP treatment at 250 and 350 MPa for 10 min during refrigerated storage [23]. No differences in lipid oxidation, estimated as thiobarbituric acid reactive substances (TBARS), were observed in smoked cod after HPP treatment for up to 600 MPa for 5 and 10 min [15].

The effects of HPP on the quality of pressurized fish meat start with protein denaturation, which drives the compaction of muscle fibers, resulting in different water-holding capacities and thus high hardness features of flesh. Protein denaturation may result in a discoloration phenomenon, and it could increase the exposure of long-chain PUFA to heme-iron, catalyzing the lipid oxidation of fish flesh. However, the high pressure values and pressure-holding time of HPP treatment, together with the chemical composition of the fish species, could lead to different changes in physicochemical parameters and even catalyze lipid oxidation according to a food matrix effect [16].

The effect of HPP on the microbial and sensory properties of seafood such as sardines has been well documented [11,12]. Most of the studies evaluated HPP treatment on already-processed sardines, such as freeze-dried [27], cold-smoked [28], or frozen ones [29].

Nevertheless, HPP is a valuable non-thermal technology, suitable for obtaining minimally processed seafood products with an extended shelf life, which may have an important economic impact on the fish market sector. A knowledge gap exists in the literature about the effect of this technology on fresh sardines, covering different quality aspects. Therefore, in this study, different HPP conditions have been evaluated on fresh sardines, considering the two main operating parameters, namely pressure value (400 and 600 MPa) and holding time (1, 2.5, 5, and 10 min). The effects of HPP were assessed by monitoring changes in physicochemical characteristics (e.g., color, texture, sensory profile) immediately after treatment (time 0) and after 7 and 14 days of cold storage at 4 °C, not only to evaluate the relation between HPP parameters and physicochemical attributes, but also the shelf-life extension.

## 2. Materials and Methods

### 2.1. Samples Handling

A total of 5 kg of sardines (*Sardina pilchardus*) were purchased from a local distributor in Vienna, Austria, and transported on ice to the pilot plant of the University of Natural Resources and Life Sciences (BOKU, Vienna, Austria). Fish were selected to have a homogenous average individual weight of 20 ± 5 g and an average length of 7 ± 2 cm. Each fish was cleaned, eviscerated, and beheaded to obtain a filet. The filets were treated later with the skin intact, and their red muscle comprised approximately 20–30% of their total muscle mass. Each sample, consisting of three fileted fish, was packed under vacuum in vacuum-sealing bags and subjected immediately to HPP treatment. A group of samples that had not been subjected to the effect of HPP was kept as a control. Three replicates for each sample were considered.

### 2.2. High-Pressure Processing (HPP) and Cold Storage

HPP treatment was conducted using a batch high-pressure processing system (U4000 Institute for High-Pressure Physics, Warsaw, Poland). A 30% *v*/*v* mixture of propylene glycol (Friogel Neo, Dehon Service SAS, Paris, France) was used as the pressure-transmitting medium. The initial temperature of the pressure vessel was set to ambient temperature. During pressure build-up (267 MPa/min), adiabatic compression heating accounted for a 2 °C/100 MPa temperature increase. Depressurization was immediate, with a pressure release time of <5 s. High-pressure treatments at 400 and 600 MPa were applied for 1, 2.5, 5, and 10 min to the fresh sardines. The chosen pressure and its holding time were determined by referring to published data, which demonstrate the effectiveness of these parameters in achieving microbial inactivation in fish [11]. After HPP, all treated and control samples, vacuum-packed, were stored in dark, refrigerated rooms at 4 °C for a maximum of 14 days. Each replicate was individually sealed in a vacuum bag to prevent repeated bag opening during analysis. Analyses were conducted immediately after treatment (time 0) and over the storage period on days 7 and 14. Seafood is highly perishable, with a shelf life of 14 days for a fresh or thawed product [30]. Based on both microbiological and sensory evaluations, the sardines kept at 4 °C surpassed the consumption threshold by day 6 [6]. Samples’ names as used in this study are expressed considering the pressure magnitude (400 or 600 MPa), holding time (1, 2.5, 5, 10 min) and storage time (0, 7, 14 days) [31].

### 2.3. Instrumental Texture Analysis

**Figure 1:**
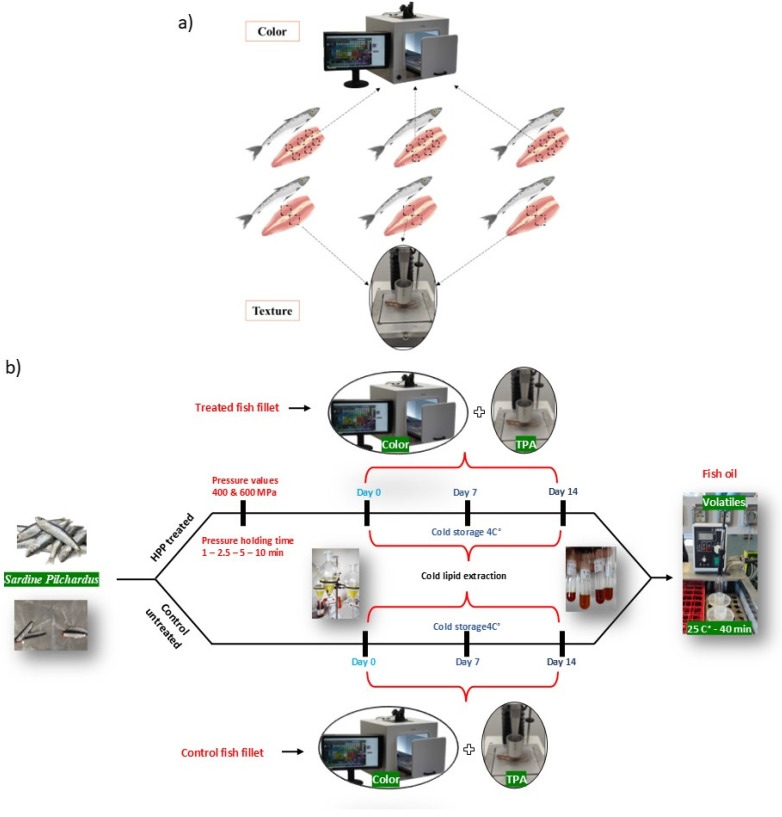
Schematic illustration. (**a**) The measured points on the surface of the sardine filet for color and texture analysis. (**b**) The experimental design. Three replicates for each sample were considered.

A texture profile analysis (TPA) for treated and control samples on day 0, 7, and 14 was carried out using a texture analyzer (Model TA-XT2i, Stable MicrosystemsTM Co., Godalming, UK) equipped with a 5 kg load cell, following the method 74–10.02 of AACCI Standard with slight modifications. The two cycles of the TPA test were carried out on the sardine filets (width of 1–2.5 cm) using a 36 mm diameter cylindrical probe (P/36R) with a height of 35 mm [32,33,34]. There were no significant differences in the size of all the filets nor the contact area between the sample and the geometry of the probe. The use of whole filets tested in different spots provides a more representative image and better reflects their natural texture. Moreover, the filets were not cut to avoid disrupting their cohesion, as they break easily, which would have introduced significant error into the data set. Adhesion to the probe, a common issue in uniaxial compression, did not occur with the fish. This was visually confirmed, as the penetration depth and filet thickness were too low to cause wall friction effects. The tests were set as follows: pre-speed at 3 mm/s; test speed at 5 mm/s, post-test speed at 5 mm/s; distance: 6 mm (30% of fish thickness); time: 0.1 s; and trigger force: 10 g. The evaluated parameters were hardness expressed in (g), chewiness (g), springiness (%), cohesiveness (%), and adhesiveness expressed in (g.s). Three fish (six filets) for each sample were used for texture analysis. For better visualization, Figure 1 shows the precise points at which data were collected for texture parameters, Figure 1a, and the experimental design for this study, Figure 1b.

### 2.4. Instrumental Color Analysis

Color measurements were carried out using a Digi-Eye^®^ system (Verivide, Leicester, UK) integrated with a D-90 Nikon digital camera (Tokyo, Japan). The results were obtained in L* (lightness), a* (redness/greenness), and b* (yellowness/blueness) as defined in the CIELAb system, and total color differences (ΔE = ([(ΔL*)^2^ + (Δa*)^2^ + (Δb*)^2^]^0.5^) were calculated. Three fish (six filets) for each sample were used, and the measurements were taken as 1 × 1 cm at three points of each filet (near the tail area; the middle part; and near the head area) as reported in Figure 1. A total of 18 readings were made for each sample and analyzed using image analyzer software (ImageJ 1.47v, National Institute of Health, Bethesda, ML, USA) as described by [31]. The instrument was calibrated on white and color standard tiles. The results were reported as an average ± standard deviation of 18 readings for each sample.

### 2.5. Analysis of Volatile Compounds

Total lipid extraction was performed at room temperature according to [35]. Briefly, each sample of sardine was homogenized with a proper volume of chloroform/methanol mixture 1:1 *v*/*v*, following the addition of aqueous potassium chloride 0.88%. The solvent was removed in a rotary evaporator after layer separation, and sardine oils were stored at −20 °C [36]. Subsequently, the effect of HPP on the lipid quality of sardines was monitored by scanning the volatile profile of sardine oil samples in GC-MS (gas chromatograph Clarus 600, Perkin Elmer, Milan, Italy) coupled with a mass spectrometer (Clarus 600 S, Perkin Elmer, Milan, Italy). Volatile compounds were collected using headspace solid-phase micro-extraction (HS-SPME) fiber (divinylbenzene/Carboxen/polydimethylsiloxane, 50/30 μm coating). For quality control, the fiber was conditioned before each daily use in the GC injection port, and a blank injection was performed to verify the absence of external compounds that might be desorbed from the fiber.

The sardine oil of treated and control samples was weighted (0.15 g) into a 4 mL screw cap vial and the fiber was inserted through the septum and left for volatile extraction for 40 min at 25 °C. Subsequently, the fiber was exposed to the hot injector port of GC for a further 10 min at 260 °C in splitless mode, with helium as the carrier gas at a flow rate of 1.2 mL/min. Volatiles were separated on DB-WAX column (60 m × 0.25 mm, 0.25 µm, J&W Scientific, Santa Clara, CA, USA). The oven temperature program was as follows: 35 °C for 4 min, raised to 120 °C at 2.5 °C/min, then set at 15 °C/min until 250 °C and held constant for 4 min; the total run was 50 min, and these conditions were adapted from those as described by [37]. The mass spectra of compounds were obtained by electron impact ionization at 70 eV and detected through full-scan mode from 30 to 400 *m*/*z*. Chromatogram data were elaborated by means of peaks using TurboMass ver. 5.4.2 software, and volatile identification was carried out by comparing their retention index, calculated using straight-chain alkanes C7-C30 (purchased from Merck, Darmstadt, Germany) to those reported in the NIST database.

### 2.6. Statistical Analysis

Experimental data of the carried analysis were subjected to the analysis of variance (ANOVA) to evaluate the significant differences (*p* < 0.05) between and within HPP-treated sardines and control ones and their interactions during cold storage. These analyses were accomplished using Statistica software (version 14.0.1.25) to monitor the changes in volatile classes, texture, and color measurements. Color parameters were statistically analyzed for control, 400, and 600 MPa (27 variables) during the storage period for each feature separated (i.e., L*, or a*, or b*) using Statistica software. Differences in texture features were monitored considering control and different HPP treatments (9 variables) independent of storage period. For color and texture data, important features, selected by ANOVA plot with a *p*-value threshold of 0.05, have been reported.

MetaboAnalyst 5.0 online platform was also employed for the visualization of principal component analysis (9 variables), fold changes, and the heatmap, which are reported to show the distribution and the variation between volatile compounds at the end of the storage period. Excel, Microsoft Office Professional Plus 2019 (version 2304), was used to build all the presented tables.

## 3. Results and Discussion

### 3.1. Effect of HPP on Texture Profile During Cold Storage

The texture of raw fish is recognized as a remarkable attribute in assessing the perceived quality of seafood, and it influences consumers’ decisions at the time of purchase [16]. Considerable positive effects of HPP on textural properties, taste, and flavor have been reported [38]. A texture profile analysis for control and HPP-treated sardine filets was performed, and texture parameters, including hardness, springiness, chewiness, cohesiveness, and adhesiveness, were monitored.

**Figure 2:**
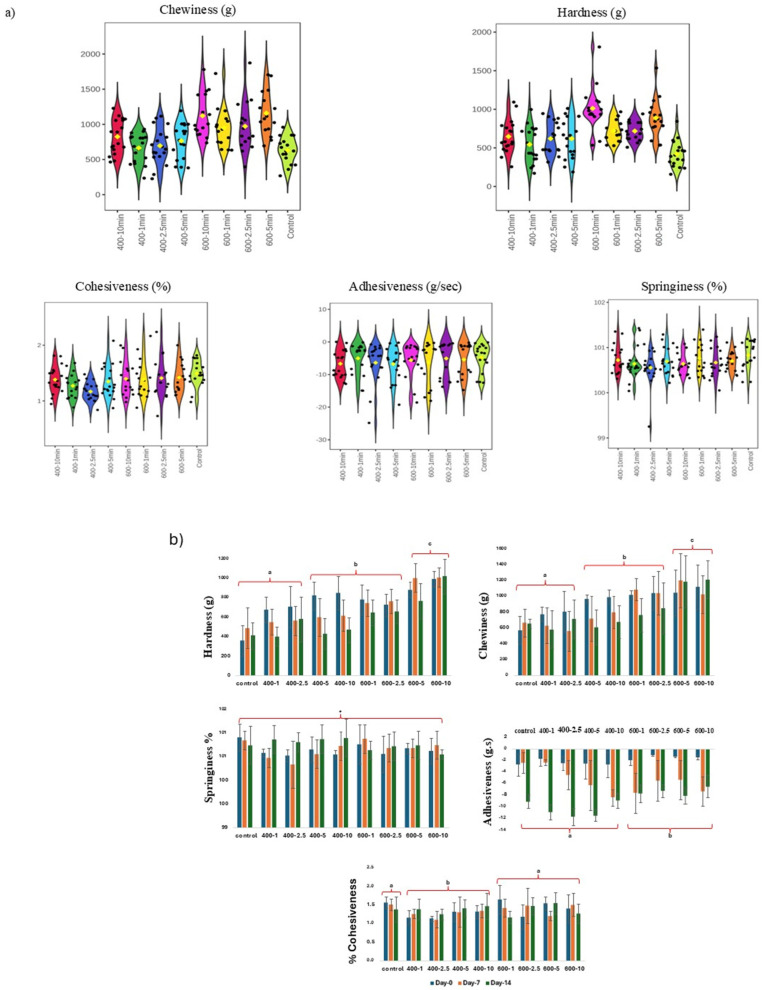
(**a**) Important features selected by ANOVA plots with *p*-value threshold 0.05. (**b**) Changes in TPA profile (hardness, chewiness, springiness, cohesiveness and adhesiveness) in sardine filets after HPP treatment and during cold storage (0, 7, 14 days), in comparison to the control. Samples are named as follows: pressure level in MPa; holding time in minutes. Three replicates for each sample were considered. Different letters indicate significant differences (*p* < 0.05) between control and HPP-treated samples independently from the storage day. * indicates no significant differences.

HPP samples exhibited a significant change in hardness and chewiness compared to the untreated samples (Figure 2a). Samples treated at 400 MPa exhibited higher values for chewiness and hardness compared to control ones. However, no significant differences were reported between various holding times at this pressure (i.e., 400 MPa), while holding the pressure of 600 MPa for 5 and 10 min significantly increased the hardness and chewiness of sardine filets (Figure 2a). These texture parameters showed considerable changes during storage as well (Figure 2b). On day 0, the hardness of the samples subjected to 600 MPa for 10 min tripled to 994.9 g compared to the control sample (358.3 g). At the end of the storage period of 14 days, the hardness of 600 MPa–10 min samples reached 1023.4 g, while chewiness was the highest in these samples, accounting for 1214. There were no significant differences observed in springiness between the studied samples (treated and control), and it remained stable throughout the tested storage period (Figure 2a). Adhesiveness was affected by HPP treatment at 600 MPa, and it increased by −1.5 g/s compared to the control −2.8 g/s (Figure 2b). Notably, adhesiveness was decreased within and at the end of the storage period for control and treated samples.

Variations in texture may be directly associated with how HPP affects proteins, as the retention of moisture in pressurized muscles during refrigerated storage is likely due to structural changes in proteins induced by high pressure, which, in turn, influence protein hydration within the muscle [39]. This includes phenomena such as protein denaturation and aggregation, modifications in actin and myosin interaction, the release of α-actinin, and the denaturation of myofibrillar proteins [16]. However, the variations may be connected to both the process parameters and the species of fish [16]. The decrease in adhesiveness observed in sardine filets treated at 400 MPa can be attributed to the denaturation of myosin, resulting in a reduction in the texture characteristics [12]. In contrast, the samples treated at 600 MPa showed an increase in adhesiveness levels, which could be explained by actin denaturation [12]. The increase in hardness and chewiness observed in this study can be elucidated by actin denaturation as well [12]. Similarly, hardness was increased after HPP treatment (600 MPa for 5 min) in Barramundi and European sea bass fish, as reported by [22]. Comparable results were reported for HPP hams prepared with farmed meagre, where high hardness and chewiness were associated with high pressure values (350 MPa for 20 min and 500 MPa for 10 min) [40]. The cohesiveness values for control and 600 MPa HPP-treated samples have no significant differences. These values decreased when treating fish filets with 400 MPa independently from the treatment duration (Figure 2b), which may be attributed to the activity of some enzymes on protein structures (the pressure stabilization of enzymes), alternating the cohesiveness values [41]. The storage period affected control samples as well as samples treated at 600 MPa, wherein values were decreased by day 14 compared to values on day 0. Tsironi et al. (2019) reported no significant effect of HPP at 600 MPa for 5 min on sea bass (*Dicentrarchus labrax*) filets in terms of cohesiveness, even after 32 days of refrigerated storage [22]. In short, HPP influences the physical properties of fish muscles; yet, this influence is considered positive for different food products, including surimi gel and salmon, as summarized by [38]. Moreover, HPP conditions at 350 and 500 MPa have been reported to improve cohesiveness in fish ham [40].

### 3.2. Effect of HPP on Color During Cold Storage

Together with the texture, color is an essential physicochemical attribute to assess the freshness of fish, and it affects the purchase actions of the consumers. Typically, the lightness (L*) of pressurized fish meat increases because of protein denaturation and an associated discoloration phenomenon; however, the variables driving color changes during HPP treatment are not fully understood yet [16].

**Figure 3:**
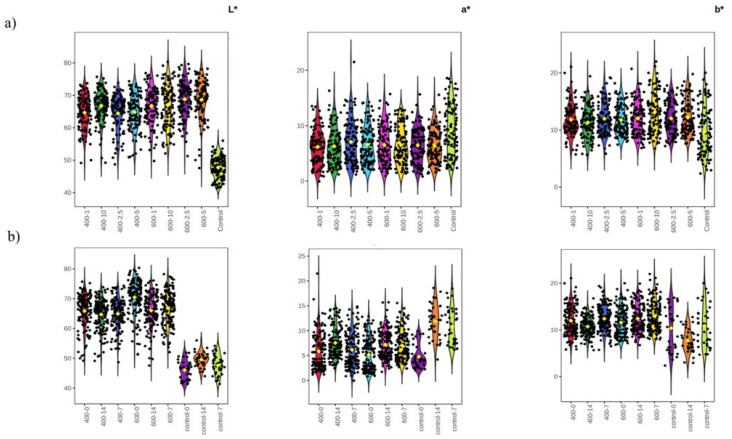
Important features for color changes selected by ANOVA plots with *p*-value threshold 0.05. (**a**) Statistical differences between control and treated samples (pressure-holding time) independently from the storage period. (**b**) Statistical differences between control and treated samples during storage.

Table 1 presents the results of color parameters L*, a*, and b* for the studied samples. L* values remained relatively stable throughout the storage period (0, 7, and 14 days) for both control and treated samples regardless of the pressure value and its holding time. Nevertheless, it is important to note that significant differences (*p* < 0.05) were reported between the control and treated samples, indicating the impact of HPP on lightness levels in sardine filets (Figure 3a). L* values for control samples ranged between 46.08 and 49.59 and increased proportionally with increasing pressure values (Table 1). Samples treated at 400 MPa had L* values varying between 63.96 and 67.21, while samples treated at 600 MPa had values from 63.53 to the highest value of 71.51 for samples with 10 min holding time (Table 1). A clear recognition of lightness in the treated samples compared to control ones can be found in Figure S1. The redness/greenness level (i.e., a* value) was decreased particularly after HPP treatment without noticeable variations within the storage period (Figure 3a). On the contrary, control samples showed an increased a* value on day 7, which was pronounced at the end of the tested storage period (a* = 11.9). It has been reported that changes in a* could be associated with oxidation [16]. Therefore, we can assume that HPP maintained the stability of a* values during the storage period. Interestingly, there were no statistically significant differences observed between treated samples in terms of yellowness/blueness level (represented by the parameter b*), while it was notably decreased in control samples on day 14 (Figure 3b). The total color changes (ΔE) tend to decrease over time during storage [38]. This trend was also observed in our samples, indicating a reduced difference in color between control samples and high-pressure-treated ones.

Comparable results were reported in albacore steaks, where L* values increased after HPP treatment for 6 min at 200 MPa [42]. A similar increasing trend was also reported in raw hilsa filets [23]. The depletion of active pigments and the coagulation of proteins are two causes of the rise in lightness seen after applying HPP. The protein coagulation changes the sample’s surface characteristics, which increases light reflection and gives it a whitish look [23,43]. The consistent stability observed in the b* rates in pressurized samples supports the assumption that sardine filets are not undergoing oxidation. This aligns with the findings of [44], who reported that the oxidation of fat in fish muscles results in a decrease in b* values. On the contrary, this phenomenon was clearly pronounced in the untreated samples. Moreover, a loss in redness value was the result of HPP treatment in hilsa filets [23]. These alterations in color characteristics caused by HPP are most likely the result of the myofibrillar and sarcoplasmic proteins becoming denatured [45].

### 3.3. Volatile Organic Profile and the Effect of HPP During Cold Storage

The volatile organic profile determines the sensory properties of fish meats, especially when secondary volatile oxidation markers are generated by the lipid oxidation reactions of the PUFA. Table S1 displays the relative percentage of the total peak area for the identified compounds, presented as an average value along with its standard deviation. Among the identified classes of volatile organic compounds, seven hydrocarbons, seven ketones, eleven alcohols, ten aldehydes, three acids, one ester, and one other compound were identified. It is worth noting that a number of the detected volatiles among aldehydes and ketones are associated with off-flavor and lipid oxidation, especially 4-heptenal, (Z)-, 2,4-hexadienal, (E,E)-, hexanal, and 2-undecanone, and they are considered secondary volatile oxidation markers [46,47].

**Figure 4:**
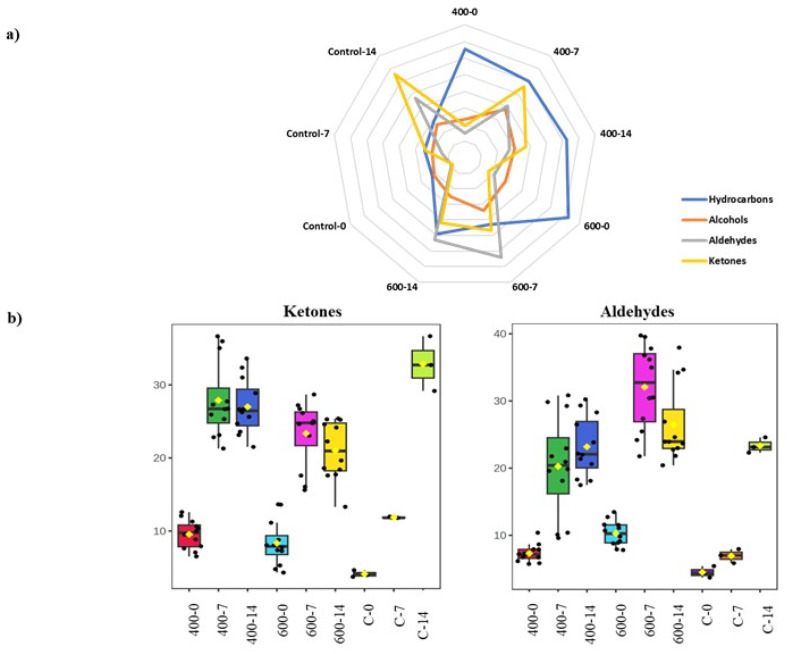
Changes in volatile classes of sardine oil in treated and untreated control samples. Samples are named as follows: pressure level in MPa; storage period in days, independently from the pressure-holding time (i.e., data for holding times were considered as one variable for each treatment). Control values are indicated by C. (**a**) The scoring radar chart displays the changes in volatile classes expressed as a relative percentage of the total peak area. (**b**) Box plots exhibiting the variation in significant differences (*p* < 0.05) in ketones and aldehydes.

Control samples were strongly affected by the end of the storage period (i.e., day 14), where they displayed the highest level of ketones (40.48%) compared to the treated ones. The changes in the volatile classes during cold storage are reported in Figure 4, and their magnitude between control and HPP-treated samples is more pronounced when the time of cold storage increases (from 0 to 14 days). Treated samples at 400 and 600 MPa on day 0 exhibited higher concentrations of hydrocarbons (alkanes and alkenes) than other volatile groups, namely aldehydes, ketones, and alcohols (Figure 4a). The pressure value (400 or 600 MPa) had a notable impact on the hydrocarbon level, particularly when the pressure was held for 10 min. Their concentrations accounted for 35.7 and 56.3% in samples treated at 400 and 600 MPa, respectively. The rate of changes was further increased by the time of storage when more volatile groups were induced on days 7 and 14. For example, alcohol, aldehyde, and ketone levels increased after HPP during storage with regard to pressure and its holding time. On day 7 of storage, samples treated at 400 MPa for 10 min exhibited concentrations of ketones and aldehydes at 35.67 and 31.27%, respectively (Table S1). In contrast, samples treated at 600 MPa for 10 min recorded concentrations of ketones and aldehyde at 28.6% and 35.91% each (Table S1). By the end of the storage period (day 14), ketones accounted for 25.34% in samples treated with 400 MPa pressure for 10 min, while samples subjected to 600 MPa pressure for 10 min exhibited ketone levels at 33.58% (Table S1). These results confirm the differences in the volatile profile for samples treated at 400 and 600 MPa. Although the storage affected the volatile classes in treated samples, this effect was more pronounced in control samples on day 14 (Figure 4b). For three minutes, the treatments of 400 and 600 MPa on human milk preserved the volatile chemicals at levels comparable to those observed in control samples of milk [48]. In meat samples treated at 400 MPa for 10 min, some alcohols and aldehydes were reduced, while other chemicals, namely 2,3-butanedione and 2-butanone, were more prevalent [49].

During the entire storage period, the levels of acids were consistently high in samples treated with 400 MPa pressure. Their concentrations were twice as high as the ones in the samples treated with 600 MPa (10.3% compared to 4.4%, respectively) for a 10 min holding time on day 14. On the other hand, esters somehow showed stability in all treated samples despite the applied pressure.

The application of HPP on sardine filets considerably affected the composition of volatile compounds. This impact on hydrocarbons was clearly expressed right after treatments. The results showed an increase in their levels compared to the control ones after the treatment. Similar findings were observed in beef sausage samples pressurized at 100 MPa for five min [50]. High concentrations of some hydrocarbons were recorded in pressurized samples compared to unpressurized ones [50]. Volatile flavor compounds including terpene hydrocarbons in Navel orange juice treated with HPP at 600 MPa for 60 s did not affect the consumers’ acceptability after storage for 12 weeks at temperatures up to 10 °C [51]. It is important to note that hydrocarbons are likely to have minimal impact on odor perception due to their high odor threshold [52,53,54,55]. Similarly, alcohols have a limited impact on flavor unless present in high concentrations [54]. Alcohols in this study were generally less abundant in all treated and control samples. Some alcohol levels detected in beef and chicken breast samples were lower after pressurization at 400 MPa for 10 min [49]. HPP treatment at 500 MPa for 30 min significantly reduced (*p* < 0.05) the concentration of alcohol in Hongqu rice-wine samples [56]. Further volatile classes were affected by storage after HPP treatment, namely aldehydes and ketones. These compound classes were significantly (*p* < 0.05) induced by pressure compared to the control after seven days of storage. This can be attributed to the high levels of PUFAs in fish flesh, which are particularly susceptible to oxidation during storage [5]. It should be noted that the fatty acid profile of different fish was not distinctively altered after HPP treatment, which makes the interpretation of volatile changes more difficult [14,57]. Atlantic salmon processed by HPP at 150 and 300 MPa, and herring (*Clupea harengus*) and minced mackerel (*Scomber scombrus*) processed at 600 MPa for 10 min did not exhibit changes in total lipid content and fatty acid composition.

On day 14 of storage, samples treated at 600 MPa had a level of aldehydes, reaching 26.8% for samples treated for 10 min. Fuentes et al. reported the enhancement of the formation of aldehyde in dry-cured ham treated at 600 MPa [58]. HPP at 600 MPa for 6 min increased the formation of aldehydes, furans, and pyrans in human milk samples [48]. On the other hand, lower pressure levels (200–300 MPa) had no significant impact on the quality of dry-cured ham products after extended cold storage, as indicated by [38]. It is possible that high pressure can release metal ions from their metal salts, which makes them ready to participate in catalyzing lipid oxidation [59]. The high content of ketones in control samples on day 14 might indicate a high level of lipid oxidation. Ketones are derivatives of lipid oxidation, and they contribute markedly to the aroma [60]. This high level of ketones can be accredited to the decreased level of hydrocarbons in the volatile profile of control samples [48]. Hence, HPP treatment at various pressures and holding times resulted in alterations in the initial volatile profile of sardine filets, particularly in the levels of ketones and aldehydes. Nevertheless, the HPP effect on their concentrations was less pronounced compared to control ones stored for 14 days in this study. This indicates that HPP treatment can modify the volatile profile of sardine filets without significantly affecting their overall concentration levels during cold storage (4 °C). Furthermore, considering the stability shown in the case of esters and the unidentified compound, we can assume that they are not products of oxidation derivatives.

### 3.4. Effect of HPP Treatment on Lipid Oxidation Markers During Cold Storage

The composition of the fish body is rich in essential fatty acids and easy-to-digest protein, making it highly exposed to oxidation. Moreover, the effect of high pressure may vary depending on this body composition. It was reported that samples treated at 300 MPa have strong catalytic oxidation levels [16]. The oxidation of lipids does not only result from pressure’s impact on lipids. Instead, it arises from the collaborative influence of oxygen and catalysts such as metal ions, proteins, or enzymes. Changes observed in sardine and blue whiting muscles in proteolytic activity can be primarily due to the impact of HPP treatment (300 MPa,) on other muscle proteins, rather than just the influence on the enzymes [61]. Mackerel muscle treated from 150 to 450 MPa for up to 5 min showed a decrease in the production of free fatty acids following HPP [62].

To assess the impact of HPP treatment on the variation in volatile compounds, focusing on oxidation markers, at the end of the tested storage period, i.e., day 14, a heatmap of the data was generated (Figure S2). The heatmap demonstrates three distinct clusters corresponding to the control samples and treated samples at 400 and 600 MPa. Samples that cluster together indicate a strong correlation and similarity among them.

**Figure 5:**
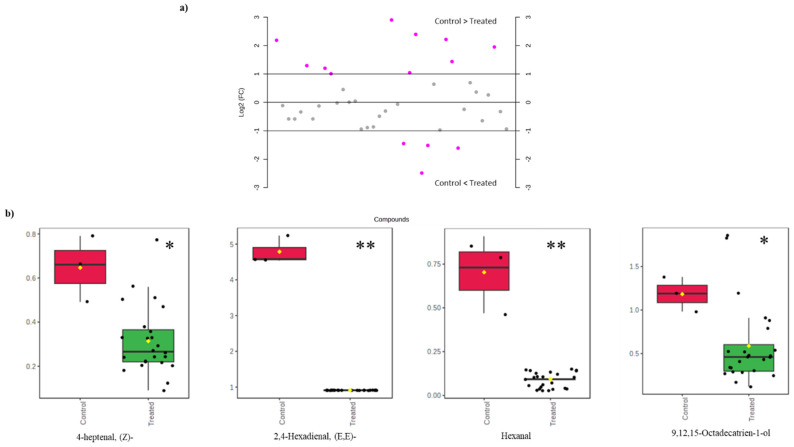
Changes in volatile compounds between HPP-treated and control samples at the end of tested cold storage (14 days). (**a**) Log2 fold change with a threshold of 3; positive values indicate predominant volatile compounds in control samples and negative ones indicate volatile compounds accumulated in HPP-treated samples. (**b**) Box plots of some discriminant compounds accumulated in control samples. Significance levels are indicated by * <0.05 and ** <0.01.

In the detected volatile compounds, control samples (C-14) exhibited the highest levels of certain compounds (Figure 5a), namely 2-pentanone, 4-hydroxy-4-methyl-, 2-nonanone, and 4-heptenal, (Z). Additionally, other volatiles were mainly and sometimes exclusively present in the control samples at the end of the storage period (Figure 5b). Particularly, hexanal and 2,4-hexadienal, (E,E)-, which can be regarded as oxidation markers, are two examples.

**Figure 6:**
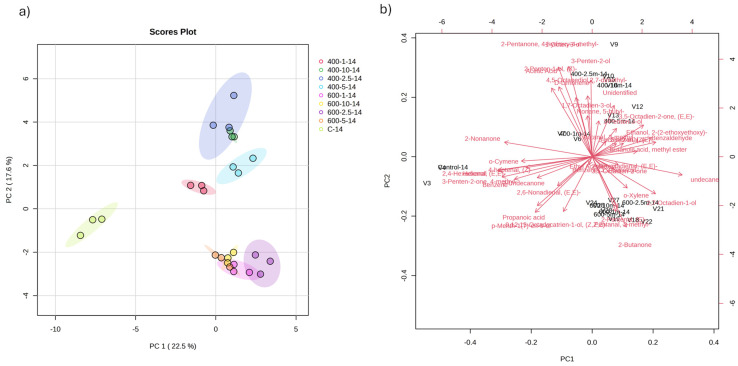
Changes in volatile compounds at the end of the tested storage period, i.e., day 14. PCA score plot (**a**) and biplot (**b**) for the volatile compound profile of sardine oil samples treated with HPP and control ones. Samples are named as follows: pressure levels in MPa; holding time in minutes; storage time in days.

A score plot of PCA analysis confirmed the differences in the volatile compositions between HPP samples and control ones at the end of the tested storage period on day 14. The variations between volatiles were further described by 40.1% by the first two principal components, PC1 and PC2, in Figure 6. However, the pressure levels of HPP treatment (400 and 600 MPa) did not have a significant effect on the composition of the volatiles’ profile at the end of the tested storage on day 14 compared to control samples that developed additional volatile compounds. PCA explained minor variations between samples with different holding times, such as samples treated at 400 MPa for 1, 2.5, 5 and 10 min as well as samples treated at 600 MPa. In the score plot of the PCA analysis (Figure 6), samples were shifted to a different quadrant when the applied pressure was increased to 600 MPa, independent of the holding time. This indicates a positive correlation between increasing the pressure and the volatile profile. The variations in ketone and aldehyde levels among the samples subjected to pressures at 400 and 600 MPa explain the observed distinction between these two groups in the PCA plot and their biplot distribution (Figure 6).

These results are in agreement with the finding of Kumar et al. (2019), who reported significant oxidation differences (*p* < 0.05) in hilsa filets treated at high pressure levels (300 MPa) compared to those treated at lower pressure levels 100 MPa [63]. Samples treated at 200 and 300 MPa had higher oxidation levels than the ones treated at 100 MPa. This phenomenon was accredited to the effect of high pressure in diminishing the electrostatic and hydrophobic forces and weakening the Van der Waals and hydrogen bonds between myofibrillar proteins [63]. Thiobarbituric acid reactive substances (TBARS) in cod samples were recorded with no differences after HPP treatment at 400 and 600 MPa for 5 and 10 min compared to untreated ones [15]. The level of some aldehydes, 1-penten-3-one, 1-penten-3-ol, 1-pentanol, and 2-pentyl furan decreased in tomato purées treated at 800 MPa for 10 min [64]. Further, a delay in lipid oxidation was reported in fish muscle treated at HPP of 150–200 MPa for 3 min [26]. Hexanal as an oxidation product of linoleic acid [65] and 2,4-hexadienal as a marker for monitoring the freshness of sardine fish [60] were two compounds distinguished for control samples, particularly towards the end of the study period on day 14. Prost et al. reported a strong increase in hexanal levels in raw sardines after 9 days of cold storage at 4 °C. Its concentration reached 62.7% of the internal standard peak area on day 9 [10].

## 4. Conclusions

HPP is an innovative non-thermal technology that can be employed in the seafood industry to extend the shelf life of products while processing them minimally. By increasing pressure values and the holding time, the hardness, chewiness, and lightness of sardine filets increased as a consequence of protein modification. Nevertheless, HPP treatment was useful in limiting oxidation reactions. Indeed, a* and b* values, usually related to oxidation, were lower in the treated samples than control ones, especially after 14 days of cold storage. More importantly, HPP-treated samples did not exhibit off-flavor compounds contrary to control ones characterized by the presence of hexenal and 2,4 hexadienal, which are known as lipid oxidation markers in fish. This study showed that high-pressure processing for fresh sardines has a high potential application in the market in the future, even for cold storage periods longer than 14 days. Given the importance of sensory and microbiological analyses of the treated sardine, future research should include detailed analyses considering these aspects.

## Figures and Tables

**Table 1:** L*, a*, and b* values of control and HPP-treated sardines at (400–600 MPa) for (1–2.5–5–10 min) during different days (0–7–14) of cold storage at 4 °C. Values are presented as an average ± standard deviation. Three replicates for each sample were considered.

	Time of Storage	L* Value	a* Value	b* Value	∆E
Control	0	46.08 ± 3.07 ^a^	4.76 ± 2.32 ^a^	10.38 ± 4.75 ^a^	0
	7	47.6 ± 3.31 ^a^	10.77 ± 3.70 ^ab^	10.87 ± 4.39 ^a^	0
	14	49.56 ± 2.41 ^a^	11.89 ± 3.38 ^b^	7.79 ± 2.49 ^b^	0
400 MPa–1 min	0	64.79 ± 5.92 ^c^	6.59 ± 3.37 ^a^	12.83 ± 3.35 ^a^	18.96
	7	65.2 ± 4.03 ^c^	4.45 ± 2.72 ^a^	11.65 ± 2.84 ^a^	18.72
	14	63.96 ± 5.00 ^bc^	7.43 ± 2.06 ^a^	10.9 ± 1.42 ^a^	15.39
400 MPa–2.5 min	0	64.49 ± 5.45 ^c^	5.88 ± 4.04 ^a^	11.43 ± 3.27 ^a^	18.47
	7	64.08 ± 5.96 ^c^	7.1 ± 3.90 ^a^	13.5 ± 2.43 ^a^	17.09
	14	64.73 ± 4.26 ^c^	8.17 ± 2.98 ^ab^	10.93 ± 1.47 ^a^	15.93
400 MPa–5 min	0	66.95 ± 5.31 ^c^	5.74 ± 3.36 ^a^	12.23 ± 2.74 ^a^	20.97
	7	63.43 ± 4.60 ^bc^	6.87 ± 3.34 ^a^	12.16 ± 2.20 ^a^	16.35
	14	64.03 ± 5.53 ^c^	7.14 ± 3.07 ^a^	10.86 ± 2.09 ^a^	15.54
400 MPa–10 min	0	67.21 ± 4.66 ^c^	5.15 ± 3.43 ^a^	11.41 ± 2.94 ^a^	21.16
	7	67.14 ± 4.13 ^c^	6.27 ± 2.66 ^a^	12.26 ± 2.52 ^a^	20.10
	14	66.05 ± 5.02 ^c^	7.4 ± 3.19 ^a^	10.18 ± 2.11 ^a^	17.26
600 MPa–1 min	0	68.72 ± 5.37 ^c^	5.34 ± 3.31 ^a^	11.46 ± 3.08 ^a^	22.67
	7	66.44 ± 4.60 ^c^	7.06 ± 3.27 ^a^	12.49 ± 2.86 ^a^	19.27
	14	65.05 ± 5.75 ^c^	7.04 ± 3.37 ^a^	12.11 ± 2.61 ^a^	16.80
600 MPa–2.5 min	0	70.74 ± 5.72 ^c^	4.76 ± 2.87 ^a^	11.01 ± 2.19 ^a^	24.67
	7	67.42 ± 4.53 ^c^	6.73 ± 2.86 ^a^	12.53 ± 3.01 ^a^	20.30
	14	67.04 ± 4.53 ^c^	7.85 ± 2.58 ^a^	12.48 ± 2.77 ^a^	18.54
600 MPa–5 min	0	69.47 ± 6.33 ^c^	5.25 ± 3.13 ^a^	11.73 ± 2.87 ^a^	23.43
	7	70.03 ± 5.03 ^c^	7.07 ± 3.24 ^a^	12.46 ± 3.13 ^a^	22.79
	14	65.88 ± 5.80 ^c^	6.94 ± 2.14 ^a^	12.32 ± 2.78 ^a^	17.65
600 MPa–10 min	0	71.5 ± 5.35 ^c^	5.33 ± 3.60 ^a^	12.05 ± 3.45 ^a^	25.48
	7	63.53 ± 5.62 ^bc^	7.31 ± 2.92 ^a^	14.27 ± 3.51 ^a^	16.65
	14	66.09 ± 5.46 ^c^	6.81 ± 2.89 ^a^	12.81 ± 3.47 ^a^	18.01

Different letters in the same column indicate significant differences (*p* < 0.05) between the control and HPP-treated samples. ∆E = [(ΔL*)^2^ + (Δa*)^2^ + (Δb*)^2^]^0.5^, where ΔL*, Δa*, and Δb* are the differences in L*, a*, and b* between the treated and control samples for each storage day.

## Data Availability

The original contributions presented in this study are included in the article/Supplementary Materials. Further inquiries can be directed to the corresponding author.

## Supplementary Materials

The following supporting information can be downloaded at: https://www.mdpi.com/article/10.3390/foods14020329/s1, Figure S1: Color changes between control samples and HPP ones (400 vs. 600 MPa) as analyzed by Digi-Eye® after HPP treatment (i.e., day 0); Figure S2: Changes in volatile compounds at the end of the storage period. A heatmap of the volatile compounds of sardine oil samples, rows (independent variables, i.e., identified volatile compounds), and columns (treated and untreated samples); Table S1: Volatile organic compounds profile detected by HSPME-GC-MS in control, untreated, and HPP-treated samples.
