# Supplementary material for: Effect of High-Pressure Processing on Color, Texture and Volatile Profile During Sardine Refrigeration
Source: Foods. 2025 Jan 20;14(2):329. doi: 10.3390/foods14020329 (PMC11764586; doi:10.3390/foods14020329)

Table S1. Volatile organic compound profiles detected by HSPME-GC-MS in control (untreated) and HPP-treated samples. Values are expressed as relative percentage of total peak area (average ± standard deviation). Experimental retention indices (RI exp) and retention indices from the literature (RI lit) are reported. Samples were named as follows [pressure value (400 or 600 MPa)–holding time (1, 2.5, 5, 10 min)–day of storage (0, 7, 14)].

N°	Compound	RI exp	RI lit	C-0	400-1-0	400-2.5-0	400-5-0	400-10-0	600-1-0	600-2.5-0	600-5-0	600-10-0
	**Hydrocarbons**											
1	Benzene	941	955	1.9 ± 1.0	2.1 ± 0.7	5.2 ± 0.4	9.2 ± 0.9	6.4 ± 1.3	4.8 ± 0.7	21.1 ± 1.1	10.8 ± 0.2	11.6 ± 1.6
2	Nonane, 5-butyl-	1064	NR	1.6 ± 0.4	4.0 ± 2	4.7 ± 0.1	10.4 ± 1.1	1.1 ± 0.5	8.1 ± 0.7	5.9 ± 0.8	4.4 ± 1.3	5.6 ± 0.2
3	Benzene, 1,3-dimethyl-	1141	1138	0.2 ± 0.3	0	0	0.4 ± 0.1	0	0.4 ± 0.1	0.2 ± 0.1	0	0.3 ± 0.1
4	o-Xylene	1185	1181	0.5 ± 0.2	0.6 ± 0.2	1.0 ± 0.5	2.8 ± 1	0.3 ± 0.1	1.9 ± 0.8	1.4 ± 0.3	0.3 ± 0.1	1.1 ± 0.4
5	D-Limonene	1199	NR	0.4 ± 0.2	0.6 ± 0.1	0.5 ± 0.3	0.5 ± 0.1	0.2 ± 0	0.8 ± 0.1	0.4 ± 0	0.6 ± 0	0.7 ± 0.2
6	o-Cymene	1273	1268	0.8 ± 0.4	0.6 ± 0.2	0.3 ± 0.1	0.1 ± 0	0.3 ± 0	0.4 ± 0.1	0.5 ± 0.1	0.2 ± 0.1	0.2 ± 0
7	undecane	1099	1100	26.0 ± 0.3	32.9 ± 0.1	32.7 ± 0.2	10.3 ± 0.9	27.4 ± 1.4	47.7 ± 1.1	28.8 ± 0.7	38.9 ± 0.6	36.8 ± 1.5
	**Alcohols**											
8	1-Penten-3-ol	1166	1158	4.9 ± 0.7	5.4 ± 1	4.4 ± 0.3	3.5 ± 0.8	5.8 ± 0.3	1.6 ± 0.8	1.6 ± 0.2	2.2 ± 0.5	2.5 ± 0.3
9	3-Penten-2-ol	1177	1183	0	0	0	0	0	0	0	0	0
10	p-Menth-1(7)-en-9-ol	1267	NR	0.1 ± 0.1	0.8 ± 0.1	0.7 ± 0.2	0.6 ± 0.2	0	0.1 ± 0	0.5 ± 0.2	0.2 ± 0.1	0.9 ± 0.3
11	9,12,15-Octadecatrien-1-ol	1279	NR	0.2 ± 0.1	0.7 ± 0.3	0.7 ± 0.2	0.7 ± 0.2	0	0.2 ± 0	0.6 ± 0.3	1.0 ± 0.1	1.0 ± 0.4
12	4,5-Octanediol,2,7-dimethyl-	n	n	0.1 ± 0.1	0	0	0.2 ± 0	0	0.5 ± 0	0.3 ± 0.2	0.2 ± 0	0.4 ± 0
13	2-Penten-1-ol, (Z)-	1329	1321	3.5 ± 0.2	2.3 ± 0.2	0.7 ± 0.4	0.7 ± 0.1	1.3 ± 0	0.9 ± 0.2	0.7 ± 0.3	0.9 ± 0.3	0.9 ± 0.1
14	1-Octen-3-ol	1459	1462	1.8 ± 0.1	1.3 ± 0.2	0.6 ± 0.1	1.4 ± 0.5	0.9 ± 0.1	0.3 ± 0.1	0.7 ± 0.3	0.9 ± 0.6	0.9 ± 0.2
15	1,7-Octadien-3-ol	1494	NR	1.1 ± 0.5	1.1 ± 0.3	0.9 ± 0.1	1.8 ± 0.4	1.3 ± 0.1	1.4 ± 0.2	1.4 ± 0.9	1.7 ± 0.6	0.8 ± 0.3
16	3-octen-2-ol, (Z)-	1499	NR	3.8 ± 0.3	3.7 ± 1	3.1 ± 1.2	3.7 ± 0.6	0.7 ± 0	1.9 ± 0.3	1.9 ± 0.1	2.4 ± 0.7	2.7 ± 0.7
17	Ethanol, 2-(2-ethoxyethoxy)-	1638	1622	0.7 ± 0.5	1.7 ± 0.2	0.6 ± 0.3	3.7 ± 0.3	0.7 ± 0.1	0.5 ± 0.2	0.5 ± 0.1	3.0 ± 0.2	1.6 ± 0.4
18	2,7-Octadien-1-ol	1697	NR	0.8 ± 0.2	0.5 ± 0.1	0.5 ± 0.4	0.8 ± 0.2	0.4 ± 0.1	0.9 ± 0.1	0.9 ± 0.1	0.8 ± 0.4	0.5 ± 0.3
	**Aldehydes**											
19	Pentanal, 2-methyl-	817	NR	0.1 ± 0.3	0	0	0	0	0	0	0	0
20	Butanal, 3-methyl-	987	NR	0.9 ± 0.1	0.8 ± 0.2	1.8 ± 0.4	0.7 ± 0.2	0.7 ± 0.4	2.7 ± 1.1	3.3 ± 0.6	0.6 ± 0.4	3.7 ± 0.9
21	Hexanal	1082	1084	0	0	0	0	0	0	0	0	0
22	2-Pentenal, (E)-	1130	1123	2.0 ± 0.4	1.8 ± 0.3	0.8 ± 0.2	0	1.9 ± 0	0	0	0.3 ± 0.2	0.8 ± 0.1
23	2-hexenal, (E)-	1220	1221	4.1 ± 0.3	1.3 ± 0.4	0.6 ± 0.4	0.3 ± 0.1	2.1 ± 0.1	0.3 ± 0.1	0.2 ± 0.1	0.3 ± 0.2	0.5 ± 0.2
24	4-heptenal, (Z)-	1244	1240	1.8 ± 0.5	0.2 ± 0.2	0.2 ± 0.1	0.1 ± 0.1	0.2 ± 0	0.1 ± 0.1	0.2 ± 0	0.2 ± 0.2	0.2 ± 0.3
25	2,4-Hexadienal, (E,E)-	1407	1407	0	0	0	0	0	0	0	0	0
26	2,4-Heptadienal, (E,E)-	1471	1479	4.9 ± 0.2	1.7 ± 0.7	0.9 ± 0.2	0.3 ± 0.2	6.7 ± 0.4	0.2 ± 0.1	0.2 ± 0	0.1 ± 0.1	0.5 ± 0.2
27	Benzaldehyde	1537	1550	1.0 ± 0.5	1.3 ± 0.3	1.0 ± 0.3	2.4 ± 0.5	0.5 ± 1	0.3 ± 0.1	1.2 ± 0.2	1.0 ± 0.6	1.1 ± 0.2
28	2,6-Nonadienal, (E,E)-	1596	NR	0.3 ± 0.1	0.2 ± 0.2	0.2 ± 0.1	0.2 ± 0	0.2 ± 0.3	0.3 ± 0.1	0.5 ± 0.1	0.4 ± 0.1	0.1 ± 0
	**Ketones**											
29	2-Butanone	905	923	1.2 ± 0.3	1.8 ± 0.2	16.7 ± 0.1	17.5 ± 0.2	13.9 ± 0.3	15.4 ± 0.1	17.8 ± 0.2	19.5 ± 0.1	9.0 ± 0
30	3-Penten-2-one, 4-methyl-	1134	1129	0.3 ± 0.3	0	0	0	0	0	0	0	0
31	2-Nonanone	1394	1388	3.3 ± 0.2	0.6 ± 0.2	0.4 ± 0.2	0.7 ± 0.2	2.1 ± 0	0.2 ± 0	0.3 ± 0.2	0.2 ± 0.1	0.3 ± 0.3
32	3,5-Octadien-2-one, (E,E)-	1534	1569	14.3 ± 0.1	11.3 ± 0.1	9.1 ± 0.3	5.7 ± 0.4	13.2 ± 0.9	0.7 ± 0.5	2.2 ± 0.7	1.7 ± 0.8	5.1 ± 1
33	3,5-Octadien-2-one	1584	NR	2.8 ± 0.1	2.7 ± 0.9	2.3 ± 1	2.9 ± 1.1	2.2 ± 0.7	0.3 ± 0.1	0.9 ± 0.5	0.9 ± 0.6	2.1 ± 0.4
34	2-Undecanone	1608	1601	0.4 ± 0.1	0.2 ± 0.1	0.4 ± 0.2	0.5 ± 0.1	0.3 ± 0.1	0.2 ± 0.1	0.3 ± 0.1	2.2 ± 0.1	0.4 ± 0.1
35	2-Pentanone, 4-hydroxy-4-methyl-	1370	1359	0.8 ± 0.5	0.1 ± 0.1	0	0	0	0.1 ± 0	0	0.0 ± 0	0.2 ± 0.1
	**Acids**											
36	Butanoic acid, methyl ester	999	993	0.3 ± 0.3	0.6 ± 0.1	1.4 ± 0.2	5.2 ± 0.2	1.0 ± 0.3	2.8 ± 0.1	2.2 ± 0.5	0.6 ± 0	1.9 ± 0.3
37	Acetic Acid	1455	1449	1.3 ± 0.5	12.6 ± 0.2	4.7 ± 0.2	6.2 ± 0.5	1.3 ± 0.6	1.2 ± 0.4	1.8 ± 0.1	1.8 ± 0.4	3.3 ± 0.8
38	Propanoic acid	1549	1547	7.6 ± 0.3	3.0 ± 1.2	1.5 ± 0.3	1.1 ± 0.1	2.3 ± 0.1	0.2 ± 0.1	0.4 ± 0.1	0.4 ± 0.3	0.7 ± 0.1
	**Ester**											
39	Ethyl Acetate	890	899	4.11 ± 0.3	1.7 ± 0.7	1.4 ± 0.3	1.6 ± 0.5	4.7 ± 0.4	0.5 ± 0.1	0.7 ± 0.2	1.0 ± 0.3	1.3 ± 0.1
40	Unidentified	1543	NR	0	0	0	3.7 ± 0	0	2.0 ± 0.3	0.3 ± 0.2	0.5 ± 0.4	0.3 ± 0.1

N°	Compound	RI exp	RI lit	C-7	400-1-7	400-2.5-7	400-5-7	400-10-7	600-1-7	600-2.5-7	600-5-7	600-10-7
	**Hydrocarbons**											
**1**	Benzene	941	955	4.1 ± 0.74	6.7 ± 1.22	0.3 ± 0.06	0.6 ± 0.05	10.7 ± 0.43	1.0 ± 0.16	6.8 ± 0.41	1.8 ± 0.08	0.9 ± 0.27
**2**	Nonane, 5-butyl-	1064	NR	2.2 ± 0.94	8.2 ± 0.81	2.4 ± 0.31	1.9 ± 1.02	0	2.5 ± 0.55	2.4 ± 0.3	2.5 ± 0.55	2.9 ± 0.33
**3**	Benzene, 1,3-dimethyl-	1141	1138	0	0.9 ± 0.23	0.6 ± 0.3	0.3 ± 0.26	0.7 ± 0.17	0.4 ± 0.22	0.7 ± 0.4	0.7 ± 0.08	0.4 ± 0.12
**4**	o-Xylene	1185	1181	0.2 ± 0.22	4.5 ± 0.22	0.2 ± 0.06	0.2 ± 0.09	0.6 ± 0.11	0.3 ± 0.09	0.4 ± 0.21	0.4 ± 0.02	0.3 ± 0.06
**5**	D-Limonene	1199	NR	0.4 ± 0.12	1.1 ± 0.41	0.4 ± 0.15	0.4 ± 0.08	7.7 ± 0.21	0.5 ± 0.21	0.4 ± 0.11	0.4 ± 0.09	0
**6**	o-Cymene	1273	1268	0.3 ± 0.18	0.4 ± 0.03	0.6 ± 0.26	0.5 ± 0.11	1.9 ± 0.34	0.8 ± 0.17	0.1 ± 0.01	0.6 ± 0.13	0.8 ± 0.12
**7**	undecane	1099	NR	56.4 ± 0.21	10.0 ± 0.96	15.1 ± 2.21	11.7 ± 0.65	16.6 ± 1	7.7 ± 1.49	4.4 ± 0.94	21.9 ± 1.39	15.2 ± 0.64
	**Alcohols**											
**8**	1-Penten-3-ol	1166	1158	1.5 ± 0.14	6.5 ± 0.67	0.9 ± 0.47	0.4 ± 0.22	1.1 ± 0.29	2.2 ± 0.23	1.3 ± 0.27	0.9 ± 0.05	2.4 ± 0.25
**9**	3-Penten-2-ol	1177	1183	0	0	0.7 ± 0.54	0.4 ± 0.13	0	1.5 ± 0.15	0.8 ± 0.27	0.6 ± 0.06	1.5 ± 0.17
**10**	p-Menth-1(7)-en-9-ol	1267	NR	0.2 ± 0.06	1.4 ± 0.27	0.2 ± 0.1	0.2 ± 0.1	0	0.4 ± 0.21	0.6 ± 0.16	0.4 ± 0.05	0.4 ± 0.06
**11**	9,12,15-Octadecatrien-1-ol	1279	NR	0.3 ± 0.15	1.1 ± 0.41	0.3 ± 0.18	0	0	0.5 ± 0.26	0.6 ± 0.28	0.5 ± 0.05	0.6 ± 0.13
**12**	4,5-Octanediol,2,7-dimethyl-	n	n	0.1 ± 0.04	0	3.4 ± 0.9	1.3 ± 0.26	5.7 ± 0.14	5.6 ± 0.53	2.2 ± 0.47	1.3 ± 0.02	5.4 ± 0.07
**13**	2-Penten-1-ol, (Z)-	1329	1321	0.7 ± 0.03	0.9 ± 0.47	0.9 ± 0.28	0.4 ± 0.03	0	1.9 ± 0.32	0.8 ± 0.36	0.6 ± 0.01	1.5 ± 0.35
**14**	1-Octen-3-ol	1459	1462	0.5 ± 0.12	1.5 ± 0.37	1.0 ± 0.4	0.4 ± 0.26	1.2 ± 0.3	1.5 ± 0.35	0.8 ± 0.07	0.3 ± 0.04	1.1 ± 0.21
**15**	1,7-Octadien-3-ol	1494	NR	1.5 ± 0.03	2.8 ± 0.22	1.4 ± 0.19	0.9 ± 0.32	0.4 ± 0.06	2.4 ± 0.78	1.4 ± 0.45	1.1 ± 0.05	2.3 ± 0.32
**16**	3-octen-2-ol, (Z)-	1499	NR	2.0 ± 0.63	5.9 ± 0.5	2.2 ± 0.03	1.8 ± 0.78	5.8 ± 0.24	1.7 ± 0.3	0.9 ± 0.23	0.7 ± 0.2	1.5 ± 0.2
**17**	Ethanol, 2-(2-ethoxyethoxy)-	1638	1622	0.7 ± 0.03	2.3 ± 0.52	1.3 ± 0.47	1.4 ± 0.16	0.4 ± 0.01	1.6 ± 0.18	1.0 ± 0.51	0.9 ± 0.15	1.7 ± 0.17
**18**	2,7-Octadien-1-ol	1697	NR	0.9 ± 0.22	1.0 ± 0.28	0.6 ± 0.26	0.4 ± 0.19	0.5 ± 0.06	0.9 ± 0.04	0.6 ± 0.41	0.8 ± 0.17	0.9 ± 0.06
	**Aldehydes**											
**19**	Pentanal, 2-methyl-	817	NR	0	0	6.3 ± 0.85	28.8 ± 0.73	0	23.5 ± 1.52	42.0 ± 1.6	34.2 ± 1.94	22.7 ± 1.84
**20**	Butanal, 3-methyl-	987	NR	0.1 ± 0.08	0.2 ± 0.08	2.2 ± 0.4	1.2 ± 0.3	0	5.6 ± 0.77	1.4 ± 0.29	0	0
**21**	Hexanal	1082	1084	2.4 ± 0.85	0	0	0	0	0	0	0	0
**22**	2-Pentenal, (E)-	1130	1123	3.4 ± 0.89	0.7 ± 0.47	0.4 ± 0.22	0.4 ± 0.23	0.6 ± 0.15	0.7 ± 0.17	0.4 ± 0.12	0.6 ± 0.1	1.1 ± 0.08
**23**	2-hexenal, (E)-	1220	1221	0.9 ± 0.31	0.7 ± 0.54	0.2 ± 0.11	0	0.7 ± 0.09	0.2 ± 0.08	0.2 ± 0.02	0.2 ± 0.04	0.3 ± 0.09
**24**	4-heptenal, (Z)-	1244	1240	0.5 ± 0.06	1.6 ± 0.3	0	0	0	0.4 ± 0.17	0.5 ± 0.09	0.2 ± 0.02	0.3 ± 0.11
**25**	2,4-Hexadienal, (E,E)-	1407	1407	0.2 ± 0.11	0	0	0	0	0	0	0	0
**26**	2,4-Heptadienal, (E,E)-	1471	1479	0.8 ± 0.3	0.8 ± 0.36	0.1 ± 0.02	0	0.4 ± 0.06	0	0.1 ± 0.01	0	0.2 ± 0.02
**27**	Benzaldehyde	1537	1550	1.1 ± 0.35	2.9 ± 0.16	1.1 ± 0.27	0.5 ± 0.38	2.8 ± 0.53	1.0 ± 0.56	0.8 ± 0.37	0.5 ± 0.14	0.9 ± 0.11
**28**	2,6-Nonadienal, (E,E)-	1596	No Ref	0.4 ± 0.08	0.2 ± 0.01	0.2 ± 0.08	0.3 ± 0.04	0	0	0	0.3 ± 0.05	0
	**Ketones**											
**29**	2-Butanone	905	923	7.1 ± 1.05	11.7 ± 0.23	1.3 ± 0.27	7.4 ± 1.11	20.8 ± 0.92	3.9 ± 0.97	12.0 ± 0.4	8.3 ± 1.03	3.3 ± 0.4
**30**	3-Penten-2-one, 4-methyl-	1134	1129	0.2 ± 0.08	0.4 ± 0.18	2.9 ± 1.08	3.8 ± 0.42	7.6 ± 0.86	4.5 ± 0.58	1.2 ± 0.35	10.1 ± 0.13	4.8 ± 0.18
**31**	2-Nonanone	1394	1388	0.4 ± 0.1	0.5 ± 0.31	0.5 ± 0.3	0.3 ± 0.14	0	0.8 ± 0.32	0.6 ± 0.2	0.4 ± 0.02	0.8 ± 0.14
**32**	3,5-Octadien-2-one, (E,E)-	1534	1569	1.7 ± 0.43	10.2 ± 1.31	1.9 ± 0.54	1.3 ± 0.41	2.8 ± 0.37	1.7 ± 0.08	1.3 ± 0.33	1.1 ± 0.11	1.7 ± 0.35
**33**	3,5-Octadien-2-one	1584	No Ref	1.0 ± 0.24	4.2 ± 0.33	1.0 ± 0.5	0.5 ± 0.52	1.7 ± 0.69	1.0 ± 0.15	0.9 ± 0.24	0.6 ± 0.13	1.0 ± 0.24
**34**	2-Undecanone	1608	1601	0.3 ± 0.15	0.3 ± 0.08	0.1 ± 0.03	20.1 ± 0.8	0.4 ± 0.06	0.3 ± 0.24	0.1 ± 0.06	0.1 ± 0.03	0.4 ± 0.15
**35**	2-Pentanone, 4-hydroxy-4-methyl-	1370	1359	0.3 ± 0.16	0	42.1 ± 1.12	8.4 ± 1.12	2.4 ± 0.46	17.0 ± 0.51	5.0 ± 1.44	5.1 ± 0.31	16.7 ± 0.5
	**Acids**											
**36**	Butanoic acid, methyl ester	999	993	0.4 ± 0.27	2.6 ± 0.23	1.2 ± 0.6	1.0 ± 0.35	0	0.5 ± 0.38	1.4 ± 0.16	0.6 ± 0.22	1.0 ± 0.17
**37**	Acetic Acid	1455	1449	2.4 ± 0.91	5.1 ± 0.61	4.0 ± 0.51	1.3 ± 0.36	6.1 ± 0.55	3.5 ± 0.42	4.6 ± 0.47	0.7 ± 0.13	3.0 ± 0.48
**38**	Propanoic acid	1549	1547	0.5 ± 0.11	0.8 ± 0.34	0.6 ± 0.28	0.2 ± 0.04	0.4 ± 0.05	0.6 ± 0.15	0.5 ± 0.11	0	0
	**Ester**											
**39**	Ethyl Acetate	890	899	3.7 ± 0.53	0.6 ± 0.09	0.4 ± 0.07	0.6 ± 0.19	0	0.4 ± 0.21	0.4 ± 0.14	0.5 ± 0.21	0.4 ± 0.04
**40**	Unidentified	1543	NR	0	1.3 ± 0.31	0.7 ± 0.11	0.4 ± 0.16	0	1.0 ± 0.37	0.5 ± 0.1	0.3 ± 0.02	1.7 ± 0.19

N°	Compound	RI exp	RI lit	C-14	400-1-14	400-2.5-14	400-5-14	400-10-14	600-1-14	600-2.5-14	600-5-14	600-10-14
	**Hydrocarbons**											
1	Benzene	941	955	4.1 ± 2.56	0.7 ± 0.1	2.5 ± 0.68	1.3 ± 0.11	1.0 ± 0.17	2.2 ± 0.81	14.0 ± 0.54	2.6 ± 0.75	1.3 ± 0.2
2	Nonane, 5-butyl-	1064	NR	2.9 ± 0.88	3.9 ± 0.31	3.2 ± 0.35	4.1 ± 0.56	1.7 ± 0.22	0.7 ± 0.07	2.2 ± 1.13	1.8 ± 0.38	1.8 ± 0.21
3	Benzene, 1,3-dimethyl-	1141	1138	0.3 ± 0.07	0.7 ± 0.1	0.2 ± 0.08	1.1 ± 0.08	0.4 ± 0.11	0.4 ± 0.21	0.2 ± 0.09	0.4 ± 0.09	0.4 ± 0.14
4	o-Xylene	1185	1181	0.3 ± 0.06	0.3 ± 0.06	0.2 ± 0.05	0.4 ± 0.11	0.2 ± 0.05	0.2 ± 0.15	1.5 ± 0.32	0.3 ± 0.03	0.2 ± 0.1
5	D-Limonene	1199	NR	0.7 ± 0.13	1.4 ± 0.06	2.0 ± 0.4	0.5 ± 0.17	1.0 ± 0.15	0.3 ± 0.05	0.3 ± 0.13	0.9 ± 0.09	1.0 ± 0.21
6	o-Cymene	1273	1268	1.0 ± 0.23	0.7 ± 0.02	1.3 ± 0.38	0.1 ± 0.02	0.9 ± 0.1	0	0.1 ± 0.05	0.8 ± 0.15	1.3 ± 0.44
7	undecane	1099	NR	10.1 ± 0.67	19.0 ± 0.02	19.5 ± 0.83	22.9 ± 1.4	11.5 ± 1.9	13.4 ± 1.25	46.4 ± 1.84	16.4 ± 1.24	16.4 ± 0.8
	**Alcohols**											
8	1-Penten-3-ol	1166	1158	1.0 ± 0.18	1.1 ± 0.12	1.9 ± 0.49	2.1 ± 0.47	9.1 ± 0.44	1.2 ± 0.39	0.7 ± 0.06	2.6 ± 0.84	1.1 ± 0.3
9	3-Penten-2-ol	1177	1183	0.5 ± 0.14	0.9 ± 0.26	1.5 ± 0.15	1.7 ± 0.51	1.0 ± 0.35	0.4 ± 0.22	0.0 ± 0.12	0.5 ± 0.02	0.4 ± 0.16
10	p-Menth-1(7)-en-9-ol	1267	NR	1.0 ± 0.16	0.4 ± 0.09	0.3 ± 0.18	0.3 ± 0.18	0.3 ± 0.12	0.7 ± 0.14	0.2 ± 0.05	0.9 ± 0.03	0.5 ± 0.1
11	9,12,15-Octadecatrien-1-ol	1279	NR	1.1 ± 0.2	0.4 ± 0.07	0.5 ± 0.17	0.5 ± 0.06	0.4 ± 0.13	1.3 ± 0.38	0.3 ± 0.13	0.8 ± 0.06	0.4 ± 0.1
12	4,5-Octanediol,2,7-dimethyl-	n	n	1.1 ± 0.69	3.7 ± 0.25	3.1 ± 0.1	2.9 ± 0.13	2.4 ± 0.35	1.4 ± 0.28	0.4 ± 0.57	1.2 ± 0.07	1.5 ± 0.43
13	2-Penten-1-ol, (Z)-	1329	1321	1.1 ± 0.13	0.8 ± 0.24	1.5 ± 0.22	1.7 ± 0.48	1.9 ± 0.25	0.4 ± 0.25	0.3 ± 0.11	0.6 ± 0.14	0.4 ± 0.14
14	1-Octen-3-ol	1459	1462	0.7 ± 0.5	1.9 ± 0.24	2.0 ± 0.45	0.9 ± 0.12	0.9 ± 0.13	0	0.2 ± 0.06	0.7 ± 0.05	0.4 ± 0.09
15	1,7-Octadien-3-ol	1494	NR	1.7 ± 0.2	2.0 ± 0.14	2.6 ± 0.43	2.9 ± 0.45	1.9 ± 0.23	1.1 ± 0.41	0.4 ± 0.13	1.7 ± 0.04	1.7 ± 0.38
16	3-octen-2-ol, (Z)-	1499	NR	1.2 ± 0.04	3.4 ± 0.27	2.9 ± 0.73	2.8 ± 0.12	2.9 ± 0.41	1.6 ± 1.1	1.1 ± 0.38	3.1 ± 0.68	2.4 ± 0.81
17	Ethanol, 2-(2-ethoxyethoxy)-	1638	1622	0.7 ± 0.16	0.9 ± 0.06	2.7 ± 0.42	1.6 ± 0.29	1.6 ± 0.22	1.2 ± 0.4	1.5 ± 0.4	1.2 ± 0.37	1.5 ± 0.26
18	2,7-Octadien-1-ol	1697	NR	0.6 ± 0.04	0.8 ± 0.21	1.4 ± 0.33	1.5 ± 0.39	0.9 ± 0.13	1.2 ± 0.39	0.3 ± 0.12	1.4 ± 0.31	1.5 ± 0.26
	**Aldehydes**											
19	Pentanal, 2-methyl-	817	NR	15.4 ± 0.7	16.1 ± 0.88	12.7 ± 0.6	15.7 ± 1.55	17.8 ± 2.25	27.3 ± 1.16	0.0 ± 1.7	17.6 ± 0.3	24.3 ± 1.97
20	Butanal, 3-methyl-	987	NR	0.7 ± 0.78	1.0 ± 0.07	1.7 ± 0.11	1.6 ± 0.13	0.8 ± 0.11	0.2 ± 0.06	0.0 ± 0.17	2.1 ± 0.54	1.1 ± 0.07
21	Hexanal	1082	1084	0.7 ± 0.22	0	0	0	0	0	0	0	0
22	2-Pentenal, (E)-	1130	1123	1.1 ± 0.31	0.2 ± 0.07	0.3 ± 0.04	1.5 ± 0.48	1.6 ± 0.22	0.4 ± 0.15	0.0 ± 0.05	1.2 ± 0.25	0.5 ± 0.19
23	2-hexenal, (E)-	1220	1221	0.1 ± 0.02	0	0.2 ± 0.02	0.3 ± 0.06	0.9 ± 0.17	0.1 ± 0.01	0	0.6 ± 0.08	0.3 ± 0.15
24	4-heptenal, (Z)-	1244	1240	1.6 ± 0.15	0.2 ± 0.04	0.2 ± 0.29	0.1 ± 0.08	0.1 ± 0.12	0.2 ± 0.02	0.1 ± 0.08	0.2 ± 0.02	0.1 ± 0.09
25	2,4-Hexadienal, (E,E)-	1407	1407	1.6 ± 0.5	0	0	0	0	0	0	0	0
26	2,4-Heptadienal, (E,E)-	1471	1479	0.1 ± 0.04	0	0.2 ± 0.04	0	1.1 ± 0.17	0.0 ± 0.04	0.2 ± 0.05	0.9 ± 0.21	0
27	Benzaldehyde	1537	1550	0.4 ± 0.07	1.0 ± 0.3	1.4 ± 0.29	1.6 ± 0.44	1.4 ± 0.3	1.2 ± 0.22	1.1 ± 0.18	0.8 ± 0.01	0.3 ± 0.08
28	2,6-Nonadienal, (E,E)-	1596	NR	0.3 ± 0.09	0.0 ± 0.02	0.2 ± 0.14	0.1 ± 0.02	0.1 ± 0.02	0.1 ± 0.04	0.2 ± 0.07	0.1 ± 0.03	0.2 ± 0.11
	**Ketones**											
29	2-Butanone	905	923	8.5 ± 0.71	8.4 ± 0.6	1.4 ± 0.17	4.4 ± 0.84	2.7 ± 0.58	29.2 ± 0.97	23.0 ± 2.4	13.7 ± 0.4	20.8 ± 1.51
30	3-Penten-2-one, 4-methyl-	1134	1129	12.3 ± 2.27	1.7 ± 0.2	1.2 ± 0.17	4.0 ± 1	2.9 ± 0.4	2.4 ± 0.77	0.1 ± 0.06	2.9 ± 0.37	3.7 ± 0.61
31	2-Nonanone	1394	1388	1.1 ± 0.22	0.8 ± 0.28	0.6 ± 0.22	0.4 ± 0.12	0.2 ± 0.07	0.1 ± 0.04	0.2 ± 0.1	0.6 ± 0.08	0.2 ± 0.03
32	3,5-Octadien-2-one, (E,E)-	1534	1569	0.6 ± 0.16	3.8 ± 0.04	2.2 ± 0.35	2.0 ± 0.36	3.3 ± 0.68	1.3 ± 0.36	1.1 ± 0.39	4.5 ± 0.44	1.2 ± 0.33
33	3,5-Octadien-2-one	1584	NR	0.7 ± 0.08	0.2 ± 0.05	0.5 ± 0.23	1.1 ± 0.46	1.4 ± 0.28	0.4 ± 0.17	0.9 ± 0.31	1.5 ± 0.24	0.5 ± 0.1
34	2-Undecanone	1608	1601	0.4 ± 0.03	0.8 ± 0.18	0.2 ± 0.11	0.2 ± 0.01	0.1 ± 0.02	0.2 ± 0.13	0.1 ± 0.02	0.4 ± 0.13	0.2 ± 0.02
35	2-Pentanone, 4-hydroxy-4-methyl-	1370	1359	16.9 ± 1.66	13.8 ± 1.13	18.3 ± 1.83	12.3 ± 0.77	14.7 ± 1.65	5.0 ± 0.91	0.0 ± 0.23	3.7 ± 0.25	7.1 ± 1.06
	**Acids**											
36	Butanoic acid, methyl ester	999	993	0.9 ± 0.27	0.9 ± 0.18	2.7 ± 0.46	1.3 ± 0.54	1.6 ± 0.23	0.3 ± 0.12	1.8 ± 0.45	1.0 ± 0.04	1.5 ± 0.29
37	Acetic Acid	1455	1449	5.1 ± 1.06	7.6 ± 0.68	4.5 ± 1.05	3.7 ± 0.8	8.8 ± 0.73	3.1 ± 0.87	1.0 ± 0.19	7.8 ± 0.43	2.5 ± 0.38
38	Propanoic acid	1549	1547	0.8 ± 0.26	0	0	0	0	0	0.1 ± 0.04	1.0 ± 0.19	0.3 ± 0.13
	**Ester**											
39	Ethyl Acetate	890	899	0.4 ± 0.08	0.2 ± 0.04	1.1 ± 0.16	0.0 ± 0.04	0.2 ± 0.04	0.2 ± 0.05	0	1.5 ± 0.11	0.5 ± 0.12
40	Unidentified	1543	NR	0.3 ± 0.1	0.2 ± 0.01	1.3 ± 0.35	0.5 ± 0.21	0.4 ± 0.09	0.2 ± 0.03	0	0.4 ± 0.12	0.5 ± 0.09

Supplementary Figures

Figure S1. Color changes between control samples and HPP ones (400 vs. 600 MPa) as analyzed by Digi-Eye® after HPP treatment (i.e., day 0).

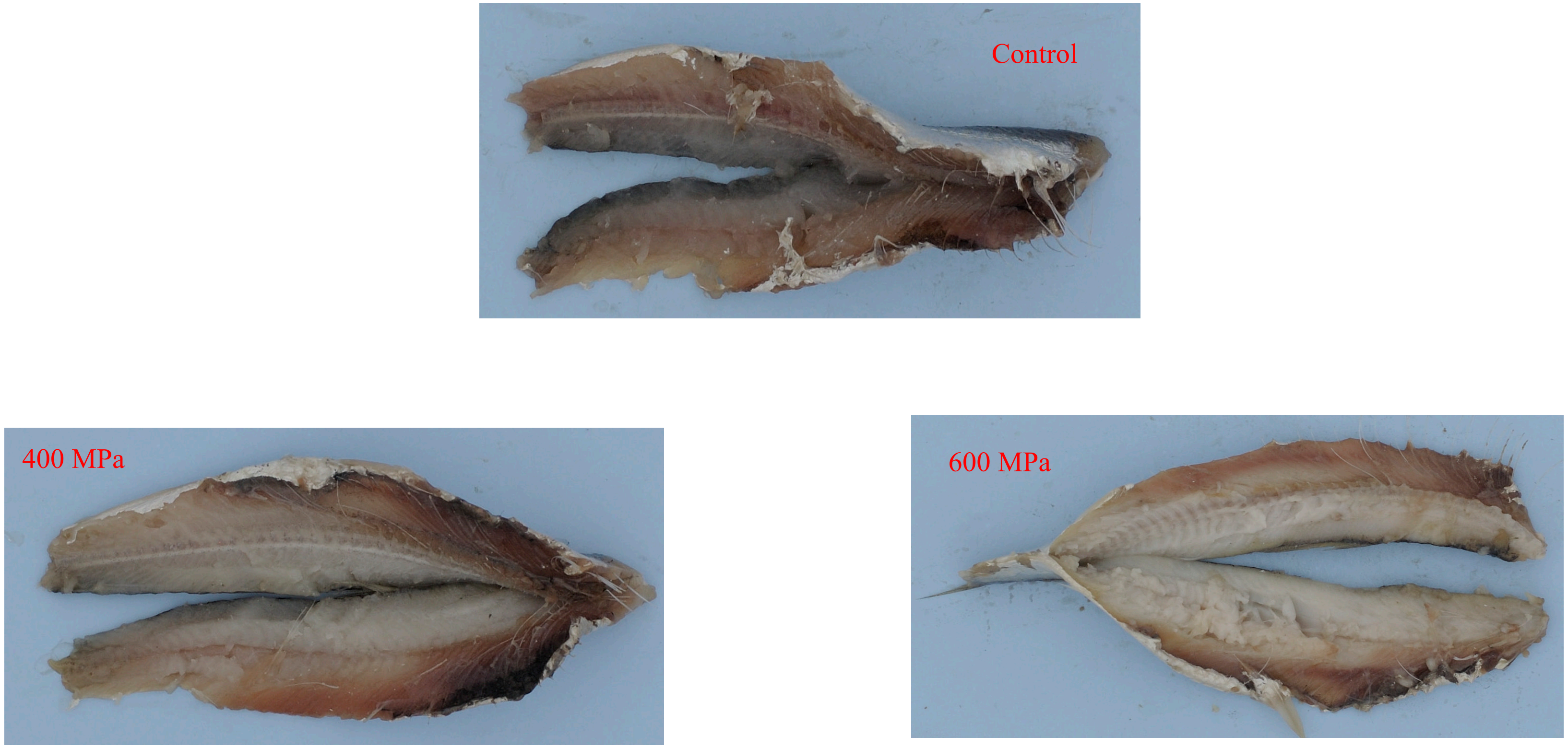

Figure S2. Changes in volatile compounds at the end of the storage period. A heatmap of the volatile compounds of sardine oil samples, rows (independent variables, i.e., identified volatile compounds), and columns (treated and untreated samples). The intensity of the red color indicates a higher percentage of the compound, while lighter shades of blue indicate lower levels of the compound.

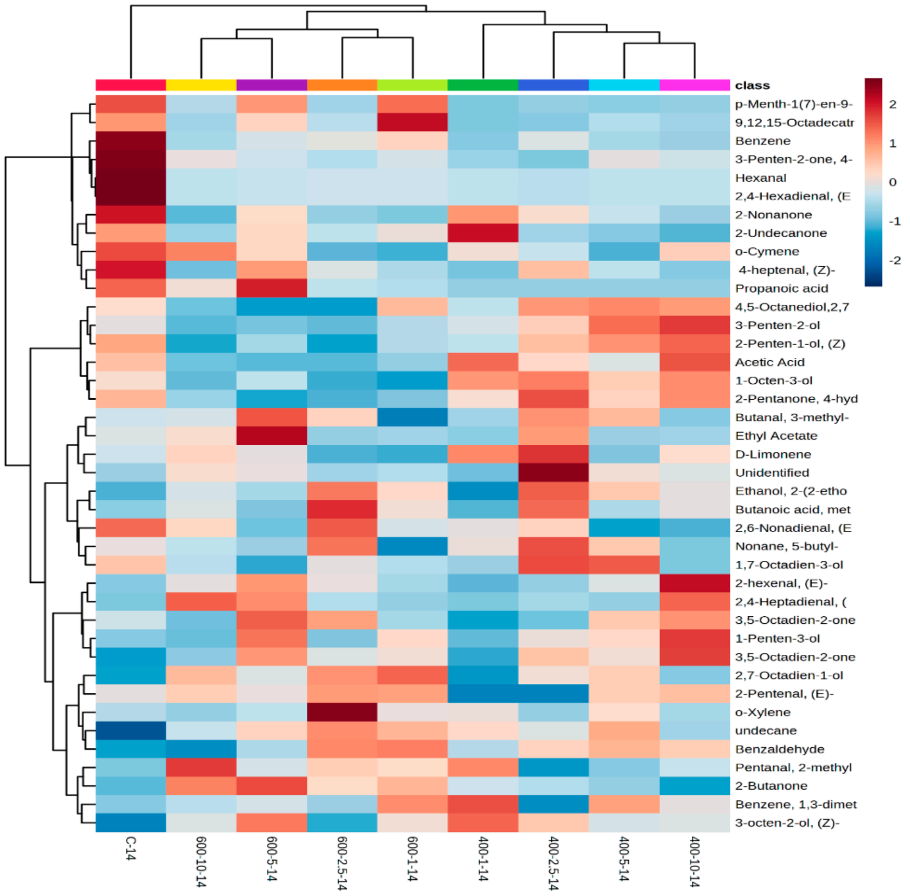

Supplement: Supplementary file 1 [file foods-14-00329-s001.zip › foods-3379666-supplementary.pdf]
